# Soluble Heparan Sulfate Fragments Generated by Heparanase Trigger the Release of Pro-Inflammatory Cytokines through TLR-4

**DOI:** 10.1371/journal.pone.0109596

**Published:** 2014-10-08

**Authors:** Katharine J. Goodall, Ivan K. H. Poon, Simon Phipps, Mark D. Hulett

**Affiliations:** 1 Department of Biochemistry, La Trobe Institute for Molecular Science, La Trobe University, Melbourne, Victoria, Australia; 2 Cooperative Research Centre for Biomarker Translation, Melbourne, Victoria, Australia; 3 School of Biomedical Sciences, University of Queensland, Brisbane, Queensland, Australia; IISER-TVM, India

## Abstract

Heparanase is a β-D-endoglucuronidase that cleaves heparan sulfate (HS), facilitating degradation of the extracellular matrix (ECM) and the release of HS-bound biomolecules including cytokines. The remodeling of the ECM by heparanase is important for various physiological and pathological processes, including inflammation, wound healing, tumour angiogenesis and metastasis. Although heparanase has been proposed to facilitate leukocyte migration through degradation of the ECM, its role in inflammation by regulating the expression and release of cytokines has not been fully defined. In this study, the role of heparanase in regulating the expression and release of cytokines from human and murine immune cells was examined. Human peripheral blood mononuclear cells treated *ex vivo* with heparanase resulted in the release of a range of pro-inflammatory cytokines including IL-1β, IL-6, IL-8, IL-10 and TNF. In addition, mouse splenocytes treated *ex vivo* with heparanase resulted in the release of IL-6, MCP-1 and TNF. A similar pattern of cytokine release was also observed when cells were treated with soluble HS. Furthermore, heparanase-induced cytokine release was abolished by enzymatic-inhibitors of heparanase, suggesting this process is mediated via the enzymatic release of cell surface HS fragments. As soluble HS can signal through the Toll-like receptor (TLR) pathway, heparanase may promote the upregulation of cytokines through the generation of heparanase-cleaved fragments of HS. In support of this hypothesis, mouse spleen cells lacking the key TLR adaptor molecule MyD88 demonstrated an abolition of cytokine release after heparanase stimulation. Furthermore, TLR4-deficient spleen cells showed reduced cytokine release in response to heparanase treatment, suggesting that TLR4 is involved in this response. Consistent with these observations, the pathway involved in cytokine upregulation was identified as being NF-κB-dependent. These data identify a new mechanism for heparanase in promoting the release of pro-inflammatory cytokines that is likely to be important in regulating cell migration and inflammation.

## Introduction

Inflammation is an important innate immune response to remove injurious stimuli and is mediated by a complex array of soluble molecules and leukocytes that are resident and/or attracted to the site of inflammation. It is well described that inflammation also contributes to many diseases including arthritis, atherosclerosis, diabetic nephropathy and cancer [1]–[6]. The migration and activation of leukocytes in inflammation are regulated by the action of chemokines and cytokines. Leukocytes also express enzymes that degrade the extracellular matrix (ECM) to aid their migration to sites of inflammation [7], [8]. Heparan sulfate (HS) is a key structural component of the ECM and basement membrane (BM) which binds an array of growth factors, chemokines and cytokines [9], [10]. The only known mammalian endoglycosidase to cleave HS is the β-D-endoglucuronidase heparanase (HPSE) [11], [12].

HPSE has been implicated in the regulation of various physiological and pathological processes. The cleavage of HS by HPSE expressed in cells such as activated leukocytes, metastatic tumour cells and proliferating endothelial cells has been proposed to facilitate degradation of the ECM/BM to promote cell migration as well as the liberation of bioactive HS-bound molecules associated with inflammation, tumour metastasis and angiogenesis [13]–[16]. HPSE also has non-enzymatic functions including cell adhesion, cellular differentiation and the activation of intracellular signaling pathways [17]–[20].

HPSE has been implicated in inflammation by regulating cellular migration via direct cleavage of the ECM and by promoting the cellular adhesion properties of migrating cells [21], [22]. When heparanase is overexpressed in transgenic mice, an enhanced response to delayed type hypersensitivity (DTH) and wound healing is observed [23], [24]. Mouse models have also highlighted the role of HPSE in type 1 diabetes (T1D) by showing that when HPSE cleaves the HS barrier around pancreatic islet BMs, induction of T1D can occur [25]. When treated with the HPSE inhibitor PI88, diabetes-prone NOD mice display preserved islet beta cell HS and reduced incidence of islet inflammation and T1D [25], [26]. Furthermore, when HPSE activity is blocked with competitive inhibitors, sepsis-induced glomerular dysfunction and inflammation are attenuated in a polymicrobial sepsis mouse model [27]. HPSE has also been implicated in the severity of atherosclerosis, as expression is increased in coronary vulnerable plaques as opposed to stable plaques and controls [28]. HPSE expression is also observed in the colon of patients suffering from Crohn's disease and ulcerative colitis [29], [30], and similarly in the synovial fluid and tissue of patients suffering from rheumatoid arthritis, but not in unaffected individuals [31]. In addition, HPSE has been implicated in central nervous system inflammatory disorders such as multiple sclerosis. Initial reports suggested that HPSE may promote experimental autoimmune encephalomyelitis (EAE), a murine model of MS, as (i) HPSE was shown to be highly expressed in infiltrating cells during EAE [32], and (ii) that the inhibition of HPSE reduced symptoms of EAE correlating with reduced lesions in the spinal cord [33], [34]. In contrast, a recent study has suggested an inhibitory role for HPSE in EAE, as administration of exogenous HPSE prior to the initiation of disease, actually resulted in ablation of symptoms [35].

Despite the proposed importance of HPSE in inflammation, its role in cytokine regulation has only recently been examined, whereby Blich and colleagues proposed that HPSE plays a direct role as a signaling molecule to stimulate the release of cytokines from primary mouse peritoneal macrophages or the mouse macrophage cell line J774 [28]. In this study, to further define the mechanism of action for heparanase in cytokine release, immune cells were treated with exogenous endotrap-purified heparanase, and cytokine release examined. HPSE was shown to stimulate the release of pro-inflammatory cytokines from human peripheral blood mononuclear cells (PBMCs) and mouse splenocytes. Interestingly, a similar trend was observed after stimulation of human PBMCs with soluble HS fragments, indicating that HPSE may be triggering the release of cytokines indirectly via the liberation of HS fragments from the cell surface and possible signaling through toll like receptors (TLRs) [36]. Indeed, this mechanism was supported by the significant reduction of cytokine release when HPSE was treated with enzymatic inhibitors and that HPSE-induced cytokine release was shown to be dependent on MyD88 and in part through TLR-4 via the NF-κB pathway. Thus, this study demonstrates that HPSE can initiate cytokine release from immune cells by generating soluble HS fragments that activate TLR-dependent pathway(s).

## Results

### HPSE promotes the release of pro-inflammatory cytokines from human PBMCs

Native HPSE was purified from human platelets [37] and passed through an endotoxin-removal column [38]. Mass spectrometry and SDS-PAGE analysis confirmed the identity of HPSE (Fig. S1A-B) and HS-cleaving enzyme activity was confirmed by a TR-FRET activity assay (Fig. S1C) [39].

**Figure 1:**
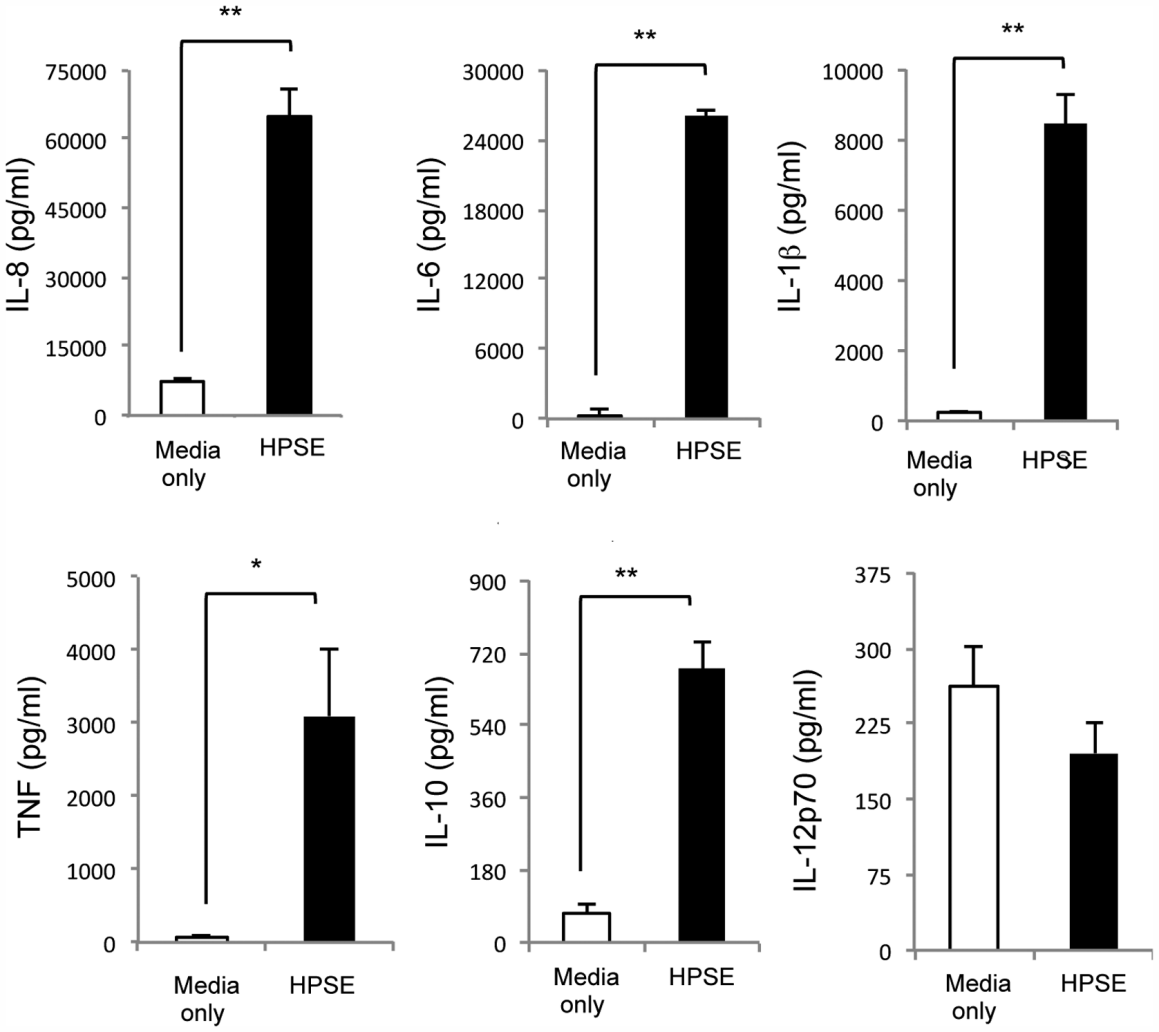
HPSE mediates release of pro-inflammatory cytokines from human peripheral blood mononuclear cells. Quantitation of IL-8, IL-6, IL-1β, TNF, IL-10 and IL-12p70 released by human PBMCs after treatment with 10 µg/mL exogenous endotrap-purified HPSE for 16 h, using a human pro-inflammatory CBA assay. Data represent the mean ±SEM of triplicate samples, representative of three independent experiments. *p = <0.05, **p = <0.001 unpaired, two-tailed Student's t-test.

To investigate the ability of HPSE to modulate the release of pro-inflammatory cytokines, human PBMCs were isolated from whole blood and treated with exogenous endotrap-purified HPSE, upon which the levels of a panel of pro-inflammatory secreted cytokines (IL-8, IL-6, IL-1β, TNF, IL-10 and IL-12p70) were determined using a cytometric bead array (CBA) assay. When treated with 10 µg/ml HPSE, a 10- to 50-fold increase in the level of cytokines in the sample supernatants at 12 h post-incubation for each of IL-8, IL-6, IL-1β, TNF and IL-10 (Fig. 1). In contrast, there was no change in levels of IL-12p70. Furthermore, the ability of HPSE to stimulate cytokine release was concentration dependent (data not shown).

### HPSE induces gene expression of pro-inflammatory cytokines from human PBMCs

**Figure 2:**
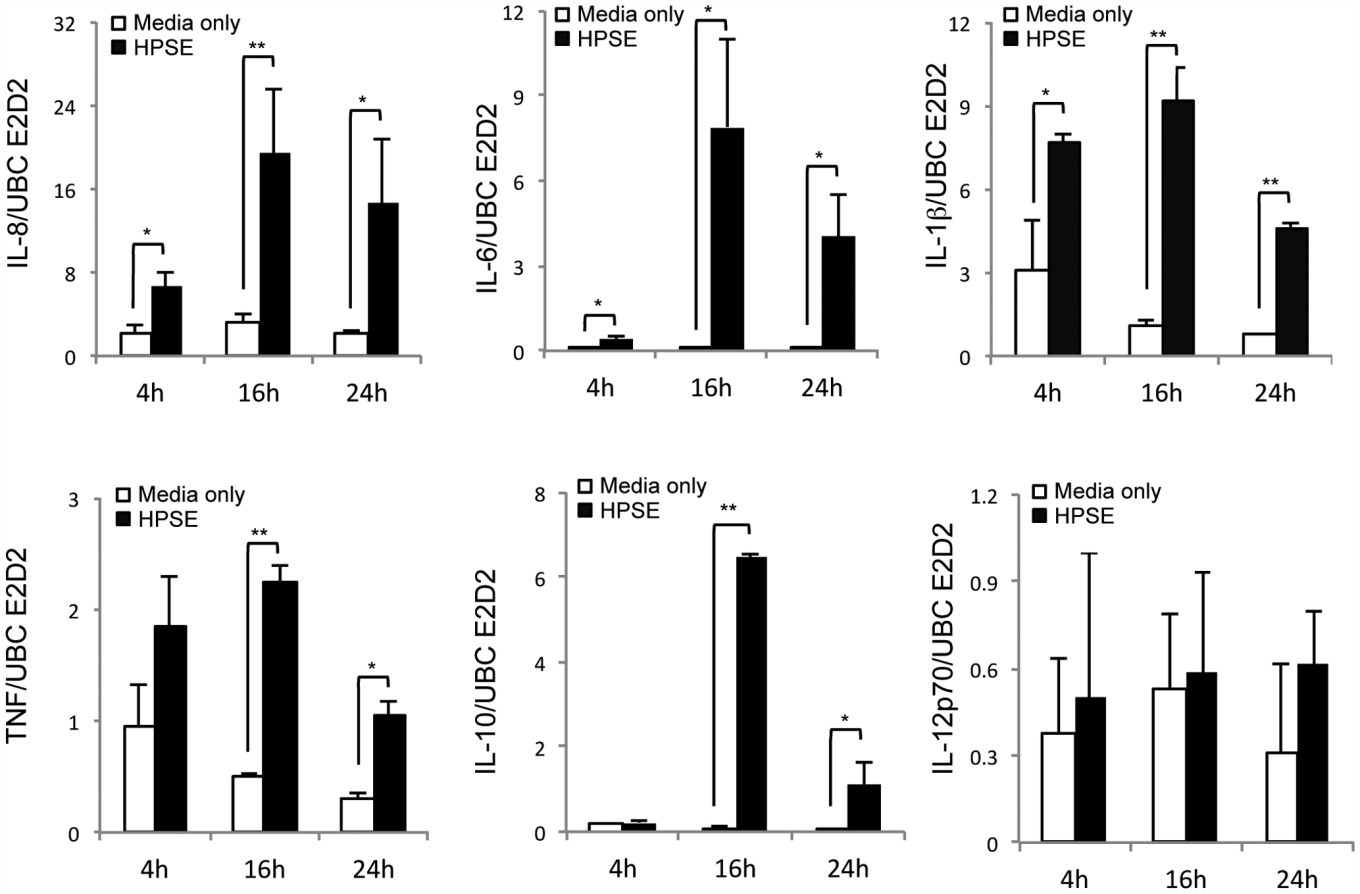
HPSE induces gene expression of pro-inflammatory cytokines from human peripheral blood mononuclear cells. Expression of IL-8, TNF, IL-1β, IL-6, IL-10 and IL-12p35 mRNA by RT-PCR in PBMCs incubated in the presence or absence of 10 µg/ml HPSE for 4, 12 or 24 h. Data are expressed as the mean level of expression normalised to *ubc e2d2*, data represent the mean ±SEM of triplicate samples, results are representative of three independent experiments. *p = <0.05, **p = <0.001 unpaired, two-tailed Student's t-test.

To determine whether increased cytokine release was associated with an upregulation of cytokine gene expression, mRNA levels of a panel of cytokines following HPSE treatment were determined by qPCR. All of the pro-inflammatory cytokines tested, with the exception of IL-12p35, showed a significant increase in mRNA levels after 4 or 16 h of HPSE treatment but this had reduced by 24 h (Fig. 2). Interestingly, the kinetics of cytokine gene expression could be divided into three groups; genes that showed (i) rapid upregulation, (ii) slow upregulation and (iii) no change. IL-6, IL-8 and IL-10 showed low expression at 4 h with peak expression at 16 h, whereas TNF and IL-1β showed high expression at 4 h that increased slightly by 16 h. IL-12p35 mRNA levels showed no significant change following HPSE treatment of cells compared with the untreated control, consistent with that observed for IL-12p70 protein levels.

### Cytokine response to HPSE is not due to endotoxin contamination

It has been well documented that LPS contamination of purified proteins can induce cytokine upregulation via signalling through TLRs [40]. Indeed, there have been many cases in which proteins were proposed to upregulate cytokine regulation, which was later found to be not reproducible due to endotoxin contamination [41]–[43].

**Figure 3:**
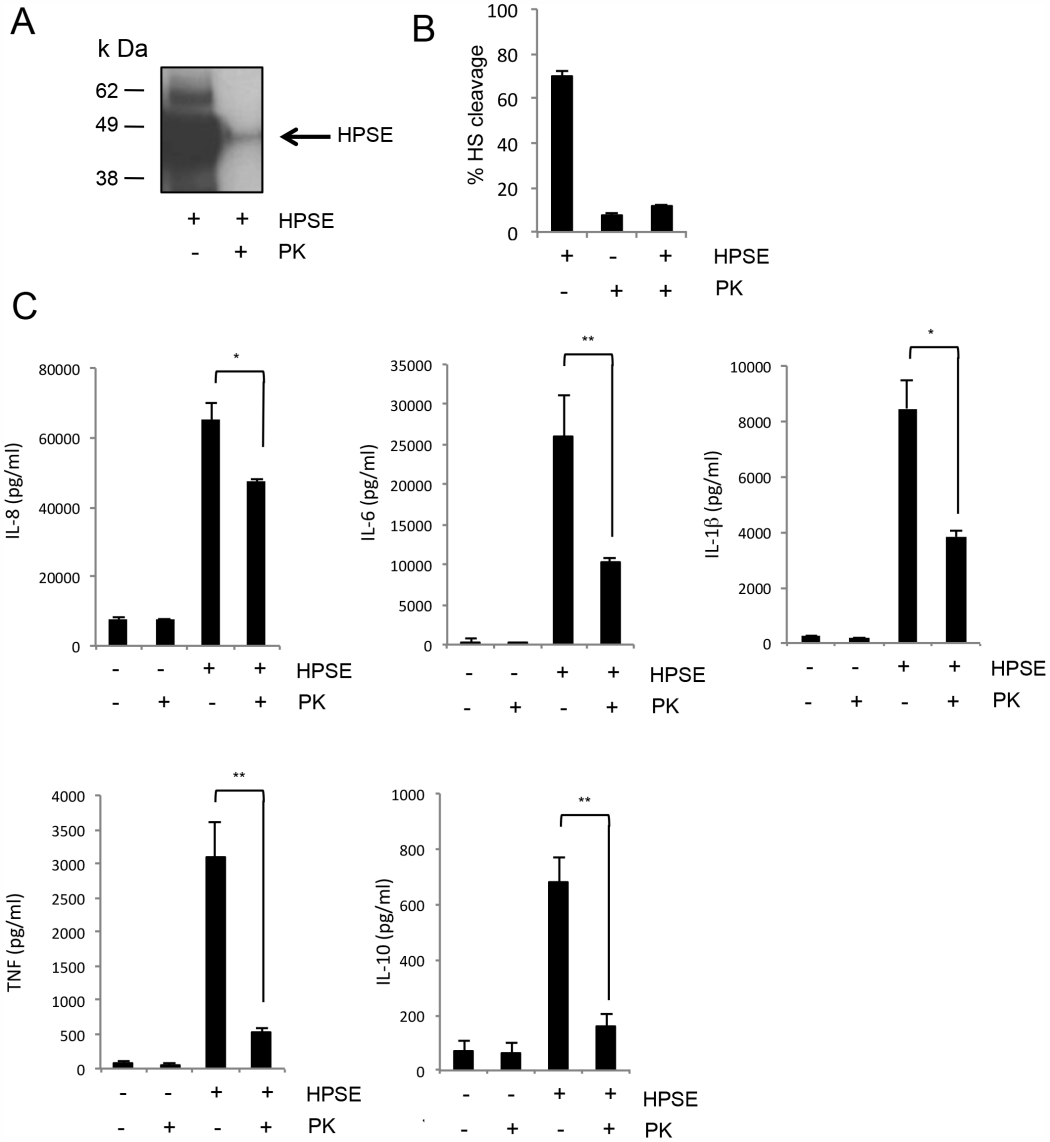
Proteinase-K treated HPSE results in a reduced cytokine response in PBMCs. HPSE (1 µg) was treated with proteinase-K agarose beads (80 µg/ml) for 30 min at 37°C before removal of proteinase-K agarose beads and SDS-PAGE analysis. (**A**) Western blot analysis for HPSE confirms that proteinase-K treatment dramatically reduced intact HPSE levels in the sample. (**B**) HPSE activity assay on identically treated samples of either HPSE alone, proteinase K alone, or HPSE treated with proteinase K. Data represent the mean ±SEM (n = 3). (**C**) Expression of cytokines after stimulation with proteinase-K treated HPSE in IL-1β, IL-6, IL-10, IL-8 and TNF in PBMCs isolated from human whole blood. Data represent the mean ±SEM of triplicate samples, results are representative of three independent experiments. *p = <0.05, **p = <0.001; unpaired, two-tailed Student's t-test.

Although HPSE was purified from human platelets and not a bacterial source in this study, to confirm that cytokine upregulation was not due to any potential endotoxin contamination, endotrap-purified HPSE, or a control of endotrap-purified DMG buffer alone, was treated with proteinase-K conjugated to agarose beads to degrade HPSE, therefore essentially removing the active protein from the sample. After treatment, proteinase-K was removed from the sample via the removal of the agarose beads. The degradation of HPSE in proteinase-K-treated samples was confirmed by Western blot analysis, as well as by a TR-FRET activity assay (Fig. 3A and B). The Western blot indicated an almost complete loss of HPSE following treatment with proteinase-K, with only minor amounts remaining (Fig. 3A). The activity assay essentially showed a complete reduction of HPSE enzymatic activity in the treated sample, with levels comparable to the background level of HPSE-free proteinase-K treated media (Fig. 3B). PBMCs were then treated with media alone, proteinase-K treated buffer, HPSE, or proteinase-K treated HPSE. For each of the cytokines examined, levels were significantly reduced when cells were stimulated with proteinase-K treated HPSE, as compared to cells treated with active, intact HPSE (Fig. 3C). However, it should be noted that proteinase-K-treated HPSE was still able to induce the release of pro-inflammatory cytokines from PBMCs compared to the control media alone (Fig. 3C), possibly due to the residual levels of intact HSPE in proteinase-K treated samples (Fig. 3A). Furthermore, proteinase-K treatment did not significantly reduce LPS-induced cytokine release (Fig. S2), ruling out any direct effect of proteinase-K on LPS. Therefore, the ability of platelet-purified HPSE to stimulate the release of pro-inflammatory cytokines is unlikely to be due to contaminating endotoxins.

### Soluble HS mediates cytokine release

**Figure 4:**
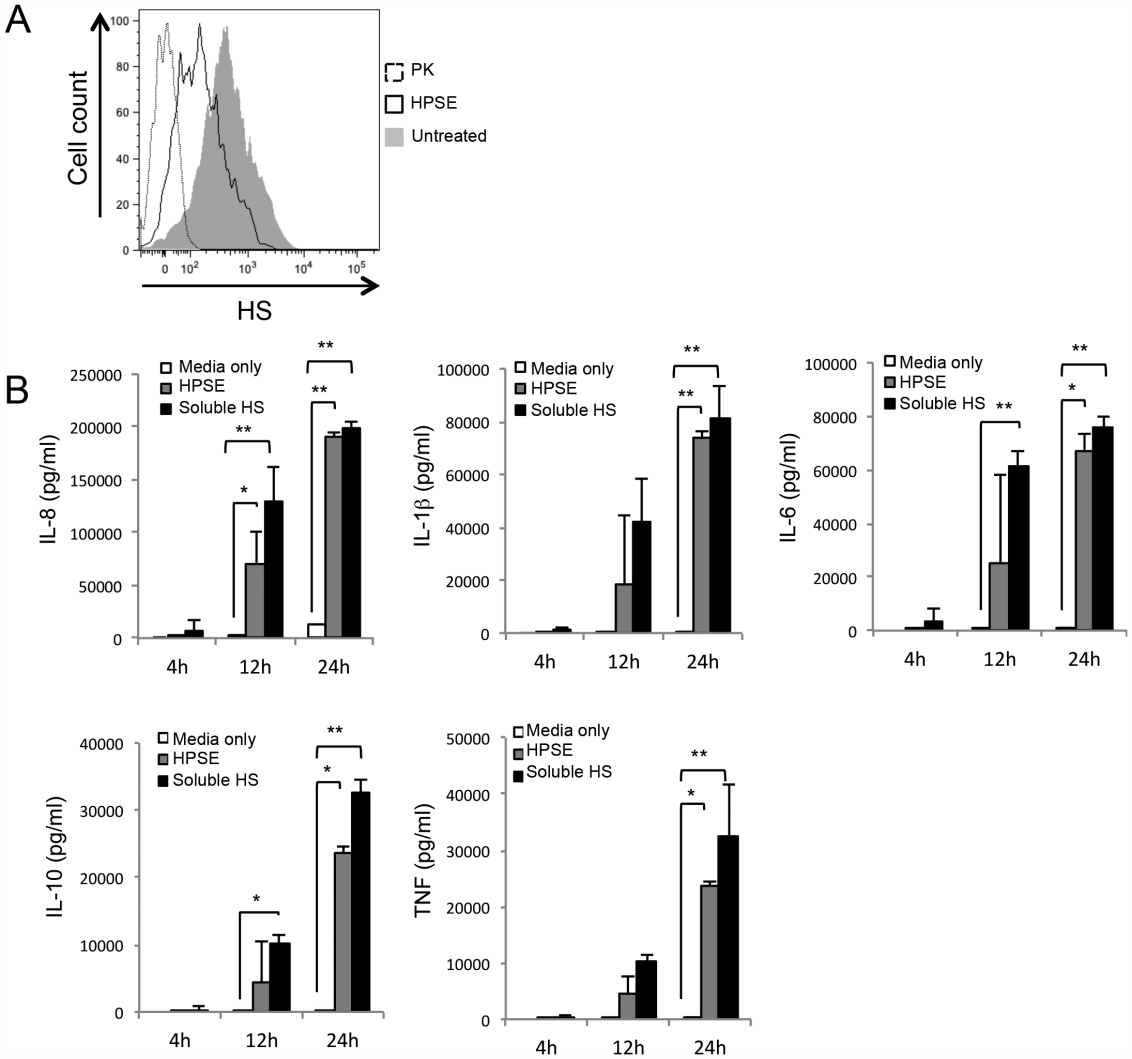
HPSE cleaves HS from the cell surface, and soluble HS leads to cytokine release. (**A**) The ability of HPSE to cleave HS in *in vitro* conditions was examined by treating cells with either the positive control of PK (100 µg/mL) or HPSE (10 µg/mL) for 1 h before staining for the presence of cell-surface HS, and analysing by flow cytometry. MFI of representative overlaid histograms of untreated (grey-filled curve), HPSE treated (empty black line), or PK treated cells (dotted line). (**B**) Endotrap-purified HS fragments (50 µg/mL) were added to PBMCs for the indicated time, before the supernatant was used in a human CBA pro-inflammatory assay to quantify cytokine release. Data represent the mean ±SEM of triplicate samples, results are representative of three independent experiments. *p = <0.05, **p = <0.001; unpaired, two-tailed Student's t-test.

A potential mechanism of action for HPSE in mediating cytokine release is via the libration of HS fragments from the cell surface that may trigger downstream signaling events. HPSE cleaves HS at regions of high sulfation, resulting in the release of 5–7 kDa soluble HS fragments [44]. Soluble HS fragments have also been shown to signal through a range of receptors on the cell surface, including Eph receptors [45] and TLRs [46]. Initially, to determine whether HPSE can cleave cell surface HS under conditions used to stimulate the release of cytokines, cells were treated with HPSE or the positive control of proteinase-K, and the level of cell surface HS was determined by flow cytometry. After 1 h HPSE treatment, the level of cell surface HS (as indicated by fluorescent intensity of HS staining) was greatly reduced, indicating that HPSE is cleaving HS (Fig. 4A). Proteinase-K treated cells also showed a significant loss of fluorescence as proteinase-K removes cell surface HS-proteoglycans. To address whether HS fragments can stimulate cytokine release, the effect of fragmented HS on human PBMCs was initially investigated. Prior to these experiments, short chain porcine HS was passed through endotoxin-removal columns (as described for HPSE). Soluble endotrap-purified HS fragments induced the release of a similar pattern of cytokines (IL-1β, IL-6, IL-8, IL-10 and TNF) from PBMCs as described for HPSE (Fig. 4B), suggesting that HPSE may be mediating the release of cytokines indirectly by cleaving HS from the cell surface, which triggers cytokine release.

### HPSE-induced cytokine release is dependent on the enzymatic activity of heparanase

**Figure 5:**
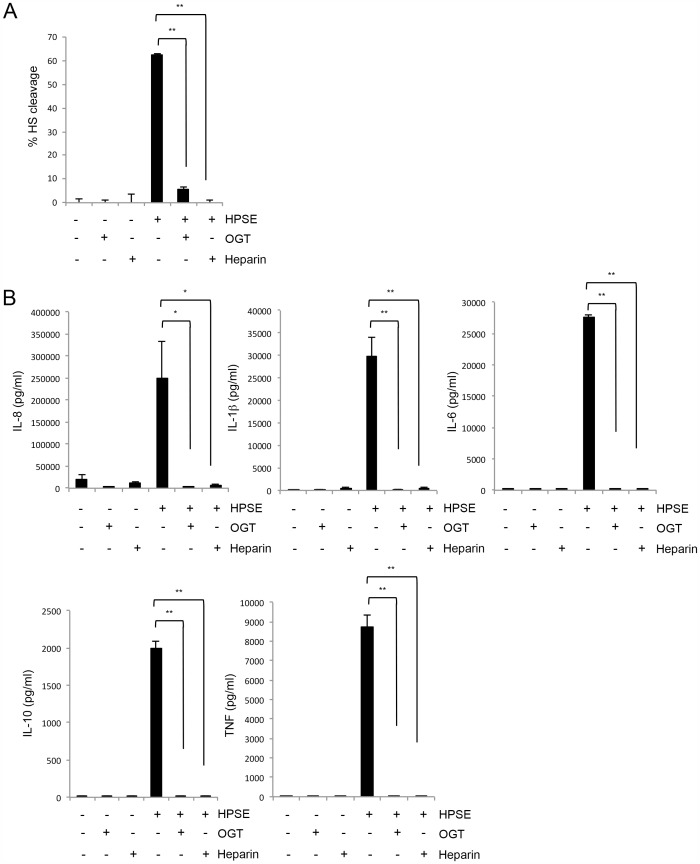
HPSE-induced cytokine release is dependent on the enzymatic activity of heparanase. (**A**) HPSE (1 ng) was treated with the enzymatic inhibitors heparin (10 ng) or OGT 2115 (20 ng) for 10 min at 37°C. HPSE activity assays were performed on treated samples of either HPSE alone, buffer or inhibitors alone, or HPSE treated with inhibitors. Data represent the mean ±SEM (n = 3). (**B**) Expression of cytokines (IL-8, IL-6, IL-1β, TNF and IL-10) by human PBMCs isolated from whole blood after stimulation with heparanase (10 µg/ml) treated with enzymatic inhibitors heparin (100 µg/ml) or OGT 2115 (200 µg/ml) for 24 h. Data represent the mean ±SEM of triplicate samples, results are representative of three independent experiments. *p = <0.05, **p = <0.001; unpaired, two-tailed Student's t-test.

To determine whether HPSE is acting in an enzymatic or a signalling manner to induce cytokine release, the HPSE inhibitors OGT 2115 [44], [47] or heparin [48], [49] were used to block the enzymatic function of HPSE. The enzymatic activity of HPSE was significantly reduced in the presence of OGT 2115 or heparin, as determined by a HPSE activity assay (Fig 5A). When treated with HPSE in the presence of OGT 2115 or heparin, human PBMCs showed significantly reduced levels of cytokine release (IL-8, IL-6, IL-1β, IL-10 and TNF) in comparison to cells treated with enzymatically active HPSE or inhibitors alone (Fig. 5B).

### HPSE-induced cytokine release is through a MyD88-dependent pathway

**Figure 6:**
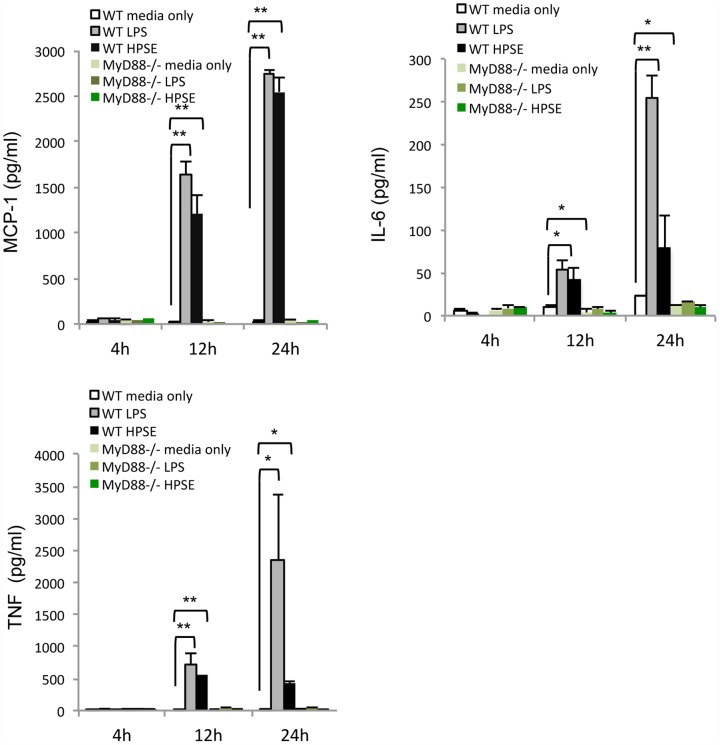
HPSE dependent cytokine release is via a MyD88-dependent pathway. Mouse splenocytes from WT and MyD88^−/−^ mice were isolated and treated with HPSE (10 µg/mL) or LPS (100 nM) for the indicated time, before the supernatant was analysed using the CBA assay for the mouse pro-inflammatory cytokines MCP-1, IL-6 and TNF. Data represent the mean ±SEM of triplicate samples, results are representative of three independent experiments. *p = <0.05, **p = <0.001; unpaired, two-tailed Student's t-test.

The TLR family plays a key role in the innate immune system and some members are known to be receptors for soluble HS [36]. Therefore, TLRs represent attractive candidates for the signalling of HPSE generated HS fragments. As MyD88 is an adaptor protein essential for signal transduction of cell-surface TLRs [50], splenocytes from MyD88^−/−^ or WT mice were isolated and treated with HPSE. Consistent with human PBMCs, in response to HPSE stimulation, WT mouse splenocytes were able to release cytokines, including MCP-1, IL-6 and TNF (Fig. 6). However, when MyD88^−/−^ splenocytes were stimulated with HPSE, the levels of these cytokines released were significantly reduced in comparison to WT (Fig. 6). To confirm in this model system that deletion of MyD88 resulted in the loss of signalling through TLRs, LPS was used as a positive control, as it evokes a strong response in cytokine release through TLR4 [51]. In contrast to WT splenocytes, MyD88^−/−^ splenocytes showed a complete lack of cytokine release when stimulated with LPS (Fig. 6).

### HPSE-induced cytokine release is partially dependent upon the TLR4 receptor

**Figure 7:**
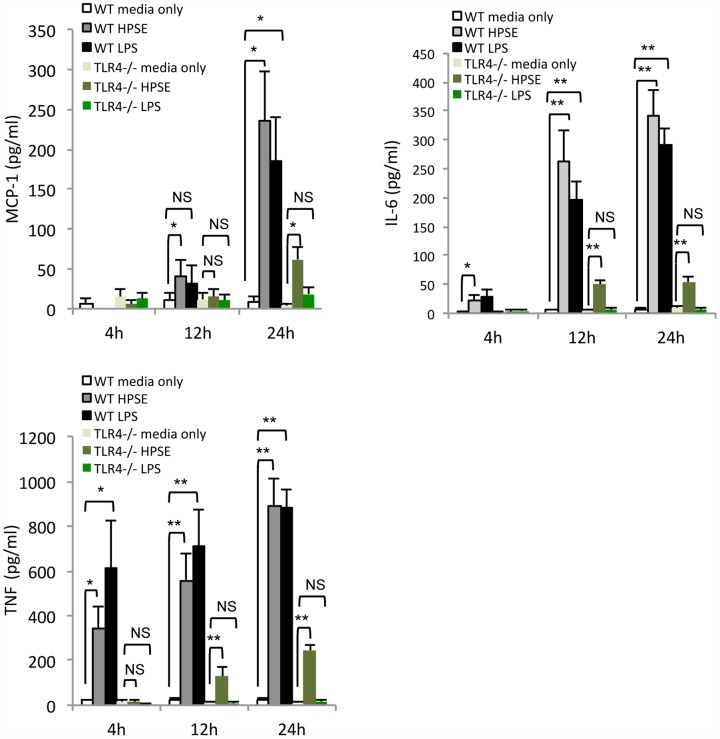
HPSE signals via TLR4 to upregulate cytokine expression. Mouse splenocytes isolated from WT and TLR4^−/−^ mice were treated with HPSE (10 µg/mL) or LPS (100 nM) for the indicated time, before the supernatant was analysed via a mouse CBA pro-inflammatory cytokine assay for MCP-1, IL-6, and TNF. Statistical analysis was performed on WT and TLR4^−/−^ HPSE treated cells, as well as TLR4^−/−^ LPS and HPSE treated cells. Data represent the mean ±SEM (n = 5). *p = <0.05, **p = <0.001; unpaired, two-tailed Student's t-test.

As soluble HS has been proposed to activate TLR4 [52], to identify the MyD88-dependent receptor involved in HPSE-mediated cytokine release, splenocytes from TLR4^−/−^ or WT mice were isolated and treated with either HPSE or the TLR4-specific agonist LPS. Interestingly, while LPS-mediated release of MCP-1, IL-6 and TNF was completely abolished in TLR4^−/−^ cells, HPSE-mediated cytokine release was significantly but not completely reduced (Fig. 7). Therefore, these data suggest that TLR4 plays an important role in HPSE-induced cytokine release, but may not be the only receptor upstream of MyD88 involved in this process.

### Extracellular HPSE stimulates the NF-κB pathway

**Figure 8:**
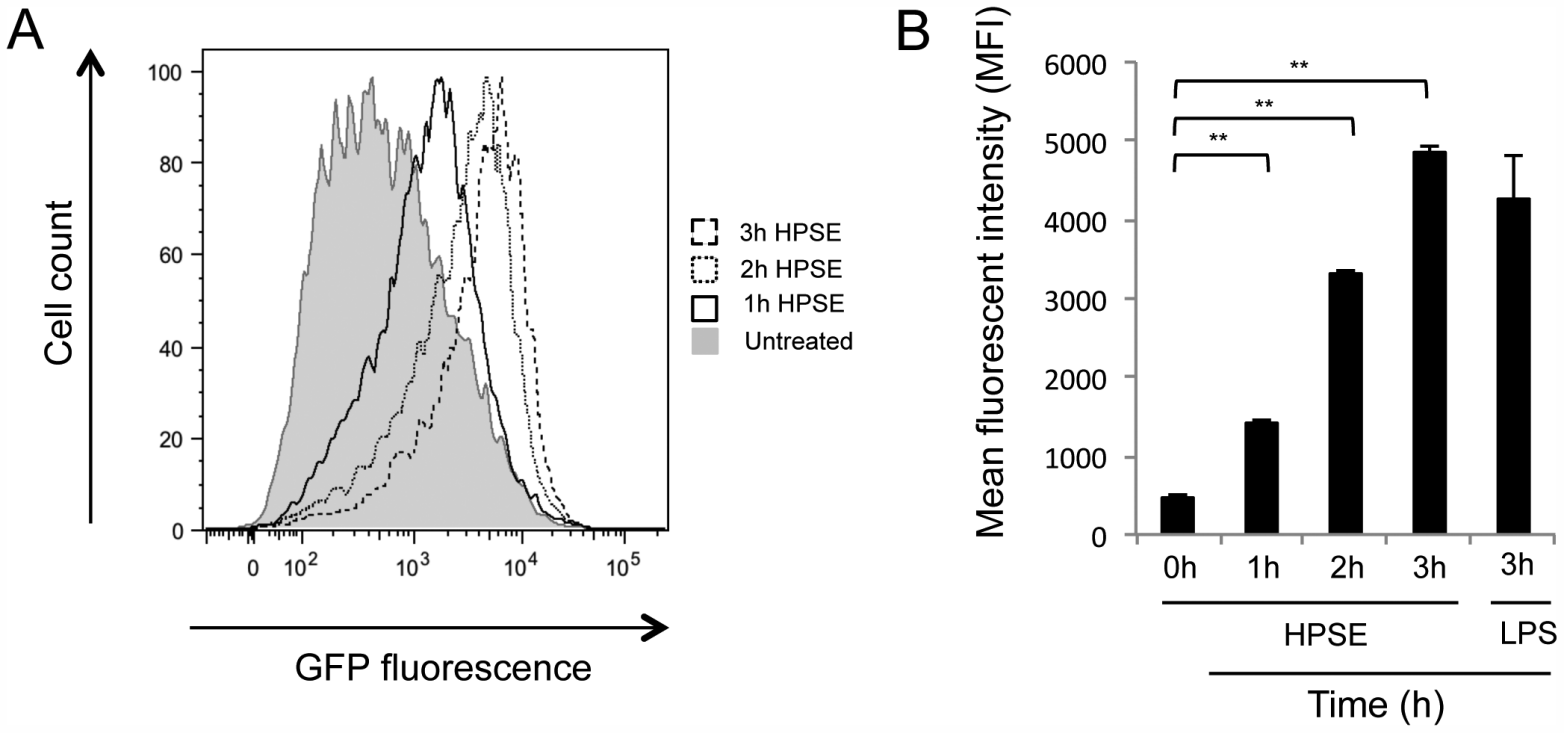
HPSE-treated cells exhibit NF-κB pathway activation. SV40T immortalised MEFS infected with pTRH1-NF-κB-GFP were treated with HPSE (10 µg/mL) or LPS (100 nM) for the indicated time and analysed by immunofluorescence flow cytometry. (**A**) Representative overlaid histograms of HPSE treated cells for 0 h (grey-filled curve), 1 h (empty black line), 2 h (dotted line) and 3 h (dashed lines). (**B**) MFI calculated from the histograms. Data represent the mean ±SEM (n = 6). * = p<0.05, ** = p<0.001; unpaired, two-tailed Student's t-test.

TLR signaling pathways classically culminate in the activation of the transcription factor nuclear factor kappa B (NF-κB) [53]. Based on the involvement of TLRs in HPSE-mediated cytokine release, SV40T immortalised mouse embryonic fibroblasts (MEFs) transfected with pTRH1-NF-κB-GFP, which exhibit GFP fluorescence if the NF-κB reporter is cleaved in either a canonical or non-canonical pathway [54] were examined. When treated with HPSE, reporter cells showed a significant increase in GFP mean fluorescent intensity (MFI), with an approximate 10-fold increase of fluorescence after 3 h incubation (Fig. 8). The kinetics of NF-κB cleavage was investigated, with a three-fold increase in GFP MFI after 1 h, which increases to a ten-fold in GFP MFI by 3 h. As a control, LPS showed maximal fluorescence at 3 h stimulation (Fig. 8). These results suggest that HPSE is acting through NF-κB to upregulate the transcription and translation of pro-inflammatory cytokines.

## Discussion

HPSE is the only known mammalian endoglycosidase with the ability to directly cleave HS, and plays an important role in ECM remodeling in a number of physiological and pathological settings. HPSE has been implicated in inflammatory disorders including arthritis, multiple sclerosis, inflammatory bowel disease and atherosclerosis [29], [31], [32], [55]. Pro-inflammatory cytokines make important contribution to all of these inflammatory diseases. This study shows that HPSE promotes the release of pro-inflammatory cytokines from both human and murine immune cells, possibly via a novel mechanism involving the generation soluble HS fragments, leading to the activation of TLR signaling.

A number of studies have implicated HPSE in the regulation of cytokine expression. The down-regulation of HPSE expression using RNA interference resulted in decreased levels of mRNA and protein of IL-8 and chemokine (C-X-C motif) ligand 1 (CXCL1) in the human malignant melanoma cell line A375 [56]. *In vivo* studies by Bitan and colleagues have shown that splenocytes isolated from mice injected with active recombinant HPSE over a period of eight days express increased levels of Th2 cytokines IL-4, IL-6 and IL-10, but decreased levels of Th1 cytokines IL-12 and TNF-α [35]. However, it should be noted that potential endotoxin contamination of recombinant HPSE would mediate a similar effect, a possibility that was not excluded in the latter study [35]. Herein, we describe that purified native human HPSE when added to *in vitro* cell cultures, induced the release of pro-inflammatory cytokines IL-1β, IL-6, IL-8, IL-10 and TNF from human PBMCs, and MCP-1, IL-6 and TNF from mouse splenocytes. It should be noted that although IL-10 is pro-inflammatory in certain settings, it also has anti-inflammatory functions [57], [58]. All experiments used enzymatically active HPSE isolated from human platelets that was endotrap-purified to remove potential endotoxin contamination. Furthermore, HPSE preparations treated with proteinase-K to remove the enzyme led to significantly reduced cytokine upregulation, indicating that HPSE and not endotoxin was responsible for the effect.

**Figure 9:**
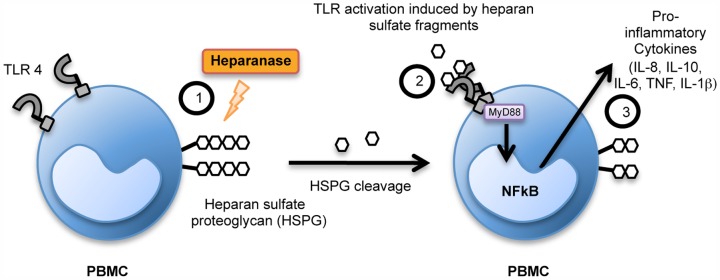
Model of cytokine upregulation in response to HPSE. Schematic model of the proposed function of HPSE in releasing pro-inflammatory cytokines. (**1**) HPSE cleaves and solubilises HS fragments from the cell surface of immune cells. (**2**) HS fragments then signal through MyD88-dependent receptors, of which TLR4 is one, and this leads to NF-κB cleavage and activation. (**3**) NF-κB-dependent upregulation of cytokine production and release, including IL-8, IL-10, IL-6, TNF and IL-1β. (**4**) Cytokines are involved in inflammatory events including chemoattraction and increased antibody production. (**5**) These events contribute to the induction of an inflammatory response.

A recent study has suggested that HPSE acts independently of its enzymatic activity to release pro-inflammatory cytokines. Murine splenocytes treated with IL-2 and the 65 kDa latent pro-form of human HPSE were shown to release cytokines [35]. However, it is important to note that this effect could be also explained by the HPSE precursor being processed in the cell system to produce an active enzyme. Herein we suggest that HPSE enzymatically cleaves HS on cell surfaces and the resultant soluble HS can in turn stimulate the release of pro-inflammatory cytokines from human and mouse primary immune cells (Fig. 9). HPSE when treated with the enzymatic inhibitors heparin and OGT 2115 resulted in the abolition of cytokine release from human PBMC. Therefore, in contrast to previous studies, these data suggest that HPSE-mediated cytokine upregulation is likely to be dependent on the HS-degrading activity of the enzyme via the generation of HS fragments, at least in part.

The ability of HS to modulate the function of cytokines has been proposed for many years, with several studies indicating that HS inhibits proteolysis of cytokines and increases the activity of cytokines by assisting their multimerisation [59], [60]. Furthermore, soluble HS has also been shown to act directly through TLRs to promote DC maturation and function as well as T-cell alloreactivity, which has the potential to increase the severity of inflammatory disorders such as graft-versus-host disease (GVHD) [61]. Blich and colleagues have recently suggested that HPSE triggers cytokine release by acting as a signaling molecule through a direct interaction with TLR4, and possibly TLR2, as an enzymatically inactive mutant of HPSE leads to the same cytokine release as WT HPSE [28]. Interestingly, in the current study, all HPSE-mediated pro-inflammatory cytokine release was significantly reduced in TLR4^−/−^ splenocytes, whereas the study by Blich and colleagues showed a reduction in TLR4^−/−^ mouse peritoneal macrophages for TNF-α levels whereas MCP-1 levels increased [28]. This raises the possibility that there are both HPSE enzymatic and non-enzymatic mechanisms involved in promoting pro-inflammatory cytokine release.

The results described herein indicate that HPSE-mediated cytokine release is MyD88-dependent and primarily involves TLR4. However, as HPSE-mediated cytokine release was not completely abolished in TLR4^−/−^ splenocytes, other receptor(s) upstream of MyD88 are also likely to make a contribution. Indeed, as indicated above, TLR2 has previously been implicated in HPSE-mediated cytokine release [28]. To precisely define the relative contributions of the TLRs in this process, additional studies are necessary using TLR2^−/−^/TLR4^−/−^ cells. We have also shown that cytokine expression in response to HPSE is transcriptionally regulated, and this result is consistent with HS fragments acting through TLRs via NF-κB. Indeed, HPSE-mediated cytokine release is reduced when cells are treated with the NF-κB inhibitor BAY117082 [28]. Furthermore, it is interesting to note that resistin has recently been identified as a new interacting partner for HPSE. Resistin has been shown to bind and signal through TLR4, leading to an increase in the pro-inflammatory protein MCP-1 [62], [63]. How the interaction of HPSE and resistin regulates pro-inflammatory cytokine production is clearly of significant interest.

In conclusion, the results presented herein suggest that HPSE can stimulate the release of pro-inflammatory cytokines by the enzymatic generation of HS fragments that in turn act through TLR-4 and another MyD88 dependent receptor, via NF-κB. This identifies a novel role for HPSE enzymatic activity in the regulation of an inflammatory response that is distinct from that previously described in facilitating the physical breakdown of the ECM to promote efficient migration of leukocytes to sites of inflammation. This study therefore broadens our understanding of HPSE function by suggesting an important multifaceted role for the enzyme in inflammation.

## Materials and Methods

### Real-time PCR (RT-PCR)

Total RNA was isolated from cells using TRI Reagent RT (Molecular Research Centre, OH) and complementary DNA (cDNA) synthesized from 1 µg total RNA using an iScript Select cDNA Synthesis Kit (Bio-Rad, CA). Complementary DNA was amplified using FastStart SYBR Green Master (Roche, Germany) with specific primers (Table 1) using the Agilent Mx300P qPCR (Stratagene, CA), with annealing temperature at 58°C and extension time of 15 seconds for all primer pairs.

**Table 1:** qPCR oligonucleotide sequences.

Gene	Forward sequence (5′ – 3′)	Reverse sequence (5′ – 3′)
UBC E2D2	GGCTCTGAAGAGAATCCACAA	TTGTAGCTTGCCAATGGAAC
IL-1β	GAAGCTGATGGCCCTAAACA	AAGCCCTTGCTGTAGTGGTG
IL-6	AGTGAGGAACAAGCCAGAGC	GTCAGGGGTGGTTATTGCAT
IL-8	CGGAAGGAACCATCTCACTG	AGCACTCCTTGGCAAAACTG
IL-10	GTGGAGCAGGTGAAGAATGC	GCCACCCTGATGTCTCAGTT
IL-12p35	CACTCCCAAAACCTGCTGAG	GGTAAACAGGCCTCCACTGT
TNF	CTGCTGCACTTTGGAGTGAT	AGATGATCTGACTGCCTGGG

### Purification of native HPSE from human platelets

Purification of human HPSE from platelets was based on a previously published method [64] with some modifications. This study was carried out in strict accordance with La Trobe Research Services Human Ethics Committee (permit number FHEC09R16) in accordance with the Australian Red Cross Blood Service agreement (number 13-05VIC-04). Expired platelets were obtained from the Australian Red Cross Blood Service (West Melbourne branch, Victoria) (http://www.donateblood.com.au/) and pelleted via centrifugation at 1600 *g* for 20 min at 20°C. The pellets were washed twice in 0.9% saline and 50–100 units of platelets were pooled for HPSE purification. The pooled platelets were resuspended in 4 volumes of sodium dimethyl glutarate buffer (15 mM sodium dimethyl gluterate (Sigma Aldrich, MO), pH 6.0, 0.5 M NaCl) and were subjected to three cycles of freeze-thawing before centrifugation at 35,000 *g* for 60 min at 4°C. The supernatant was removed and stored at 4°C before the platelets were freeze-thawed and pelleted twice more, pooling the three supernatants.

The supernatant, containing 0.2% Triton X-100 and 1 mM CaCl_2_/MnCl_2_, was passed through a concanavalin A-Sepharose (GE Healthcare, UK) column before eluting with 20% (w/v) methyl α-D-mannopyranoside and passing through a Zn^2+^-chelating sepharose column (GE Healthcare) connected in series to a Blue A-agarose column (GE Healthcare) at 4°C and washing with 0.5 M then 0.8 M NaCl. The Blue A-agarose column was then connected in series to an octyl-agarose column (GE Healthcare) and the protein was eluted from the Blue A-agarose column with 2 M NaCl and passed through the connected column. The eluted protein was concentrated in a YM-30 centrifugal filter (Amicon Biosciences) followed by further concentration in a centricon YM-30 centrifugal filter (Amicon).

### Endotoxin removal

Purified native HPSE and soluble HS was passed through an endotrap red column (Hyglos GmbH, Regensburg, Germany) as per manufacturer's specifications, collecting 100 µl fractions. To determine the concentration of HPSE in each sample, a BCA assay and a quantified Western blot were performed on the fractions. All other chemicals and samples were bought and maintained in an endotoxin free environment.

### HPSE enzymatic activity assay

HPSE activity was detected via a time-resolved fluorescence energy transfer (TR-FRET)-based assay (Cisbio, MA) according to manufacturer's instructions. For inhibitor studies, 1 ng HPSE was incubated with either OGT 2115 (20 ng) or heparin (10 ng) for 10 min at 37°C. The HPSE-treated inhibitor samples, 1 ng of untreated HPSE or proteinase-K treated HPSE were diluted in HPSE dilution buffer (20 mM Tris-HCl, 0.15 M NaCl, 0.1% CHAPS, pH 5.5) before being diluted 1∶1 with pure H_2_O and combined with the substrate Biotin-HS-Eu(K) (0.7 µg/ml Biotin-HS-Eu(K), 0.2 M NaCH_3_CO_2_, pH 5.5; Cisbio, MA). The resulting reaction was incubated at 37°C for 2 h. XL665-conjugated Streptavidin (1 µg/ml Streptavidin-XL665 (Cisbio, MA), 0.1 M NaPO_4_, pH 7.5, 1.2 M KF, 0.1% BSA, 2.0 mg/ml heparin) was then added and the reaction incubated in dark for 16 h at room temp. Excitation of 315 nm and emission of 620 nm and 680 nm was then measured. The percentage of HS degradation was calculated in relation to FRET positive or negative samples (i.e. presence of absence of XL665-conjugated Streptavidin, respectively, in the absence of HPSE). HPSE activity was measured with a SpectroMax M5^e^ spectrophotometer (Molecular Devices, CA). To determine activity per protein concentration, the final percentage of HS degradation was divided by the absolute amount of lysates assayed.

### Cytometric bead array assay

Levels of soluble cytokines were measured using a cytometric bead array (CBA) human inflammation kit or mouse inflammation kid (BD Biosciences) according to the manufacturer's instructions, using supernatant generated from stimulation of 2×10^5^ cells/well in 96 well plates. For inhibitor studies, HPSE (10 µg/ml) was treated with OGT 2115 (200 µg/ml) or heparin (100 µg/ml) for 10 min at 37°C before being added to cells. Samples were analysed by flow cytometry using a FACSCalibur, and analysed with FCAPArray Software (BD Biosciences).

### Purification of PBMCs

Whole blood was diluted 1∶1 with sterile pyrogen free PBS (Gibco) and layered onto Ficoll-Paque PLUS (GE Healthcare, Buckinghamshire, UK) before separation via centrifugation at 750 *g* for 30 min at room temperature, in the absence of brakes. After separation of layers, the PBMC layer was removed from directly above the Ficoll and washed three times in 0.1% BSA/PBS. Cells were resuspended in 0.1% BSA/RPMI and seeded into a microplate.

### Western blotting

Western blot analysis was carried out as described previously [39] using a rabbit anti-HPSE Ab (Hpa-1; 1∶3000; InSight Biopharmaceuticals, Israel) followed by HRP-conjugated anti-rabbit Ig (1∶10000; GE Healthcare).

### Proteinase-K treatment of HPSE

Proteinase-K-agarose beads (Sigma Aldrich) were suspended in endotoxin free PBS (Ca^2+^- and Mg^2+^-free, Life Technologies, CA) to a concentration of 80 mg/ml. Endotoxin-free HPSE or PBS was treated with proteinase K-agarose beads for 1 h at 37°C. The samples were transferred immediately to ice, before the proteinase-K beads were removed via centrifugation at 200 *g* for 3 min at 4°C. Proteinase-K treated HPSE or DMG buffer control were stored at 4°C until use in the human pro-inflammatory cytokines assay.

### Spleen cell purification and stimulation

Spleens from pathogen-free Balb/c wild-type, MyD88^−/−^ or TLR4^−/−^ mice [51], [65] were isolated and passed through a 100 µm cell strainer into 10 ml DMEM (Life Technologies). Cells were washed twice with DMEM before being resuspended in endotoxin-low 0.1% BSA/RPMI and seeded into a microplate. This study was approved by the La Trobe University Animal Ethics Committee (ethics approval number AEC11-34).

### Heparan sulfate binding studies

Raw macrophage 264.7 (ATCC) cells were removed from the flask with Versene (0.5 mM EDTA), washed twice with PBS and resuspended in 0.1% BSA/PBS at 4°C. Cells were incubated with mouse anti-HSPG Ab (Clone F58-10E4; Seikagaku, Tokyo, Japan) for 30 min on ice before washing twice and incubating with a secondary PE-conjugated anti-IgM Ab (eBiosciences). Cells were washed twice before being subjected to flow cytometry on a FACSCanto.

### NF-κB-GFP stimulation assay

SV40T immortalised MEFS infected with pTRH1-NF-κB-GFP (System Biosciences (TR503PA-1) were a kind gift from David Vaux and James Vince (WEHI). Cells were cultured in an endotoxin-free environment before being seeded into a 24 well plate and treated with either 10 µg/ml HPSE or 100 nM LPS. Cell fluorescence was measured on a FACSCanto, gating out cell debris, and the mean fluorescent intensity (MFI) was calculated.

## Supporting Information

Figure S1 **HPSE purification and quality assurance.** (**A**) HPSE purified from human platelets was subjected to SDS-PAGE and either stained with Coomassie or transferred to nitrocellulose and probed with an anti-HPSE antibody. (**B**) Purified human HPSE was reduced and alkylated before digestion with trypsin and analysis by MALDI-TOF-TOF-MS. The measured masses were assigned to HPSE sequence and coverage of 63% was achieved. The black bars represent the identified peptides in the human HPSE sequence. (**C**) HPSE activity assay of 1 ng purified HPSE, or buffer control. Data represent the mean ±SEM (n = 3).
(TIF)

Figure S2 **Proteinase-K does not significantly abolish LPS-induced cytokine release.** LPS was treated with proteinase-K agarose beads for 30 min at 37°C before removal of proteinase-K beads. Expression of cytokines after stimulation with proteinase-K treated LPS in IL-8, IL-1β, IL-6, IL-10 and TNF in human PBMCs. Data represent the mean ±SEM of triplicate samples, results are representative of three independent experiments). * = p<0.05, ** = p<0.001; unpaired, two-tailed Student's t-test.
(TIF)
