# Supplementary figures and images for: Soluble Heparan Sulfate Fragments Generated by Heparanase Trigger the Release of Pro-Inflammatory Cytokines through TLR-4
Source: PLoS One. 2014 Oct 8;9(10):e109596. doi: 10.1371/journal.pone.0109596 (PMC4190175; doi:10.1371/journal.pone.0109596)

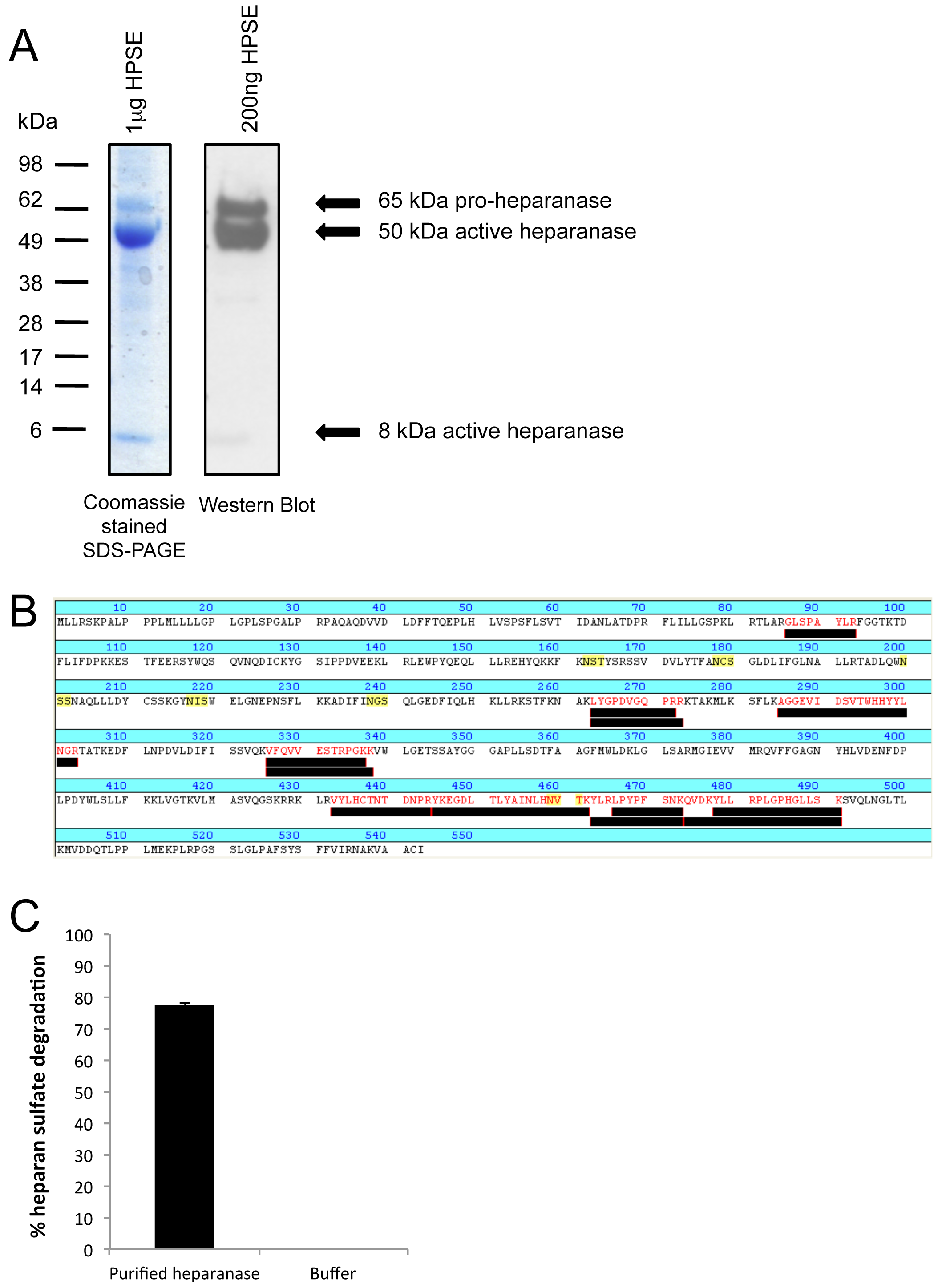

Supplement: Figure S1 — HPSE purification and quality assurance. (A) HPSE purified from human platelets was subjected to SDS-PAGE and either stained with Coomassie or transferred to nitrocellulose and probed with an anti-HPSE antibody. (B) Purified human HPSE was reduced and alkylated before digestion with trypsin and analysis by MALDI-TOF-TOF-MS. The measured masses were assigned to HPSE sequence and coverage of 63% was achieved. The black bars represent the identified peptides in the human HPSE sequence. (C) HPSE activity assay of 1 ng purified HPSE, or buffer control. Data represent the mean ±SEM (n = 3). (TIF) [file pone.0109596.s001.tif]

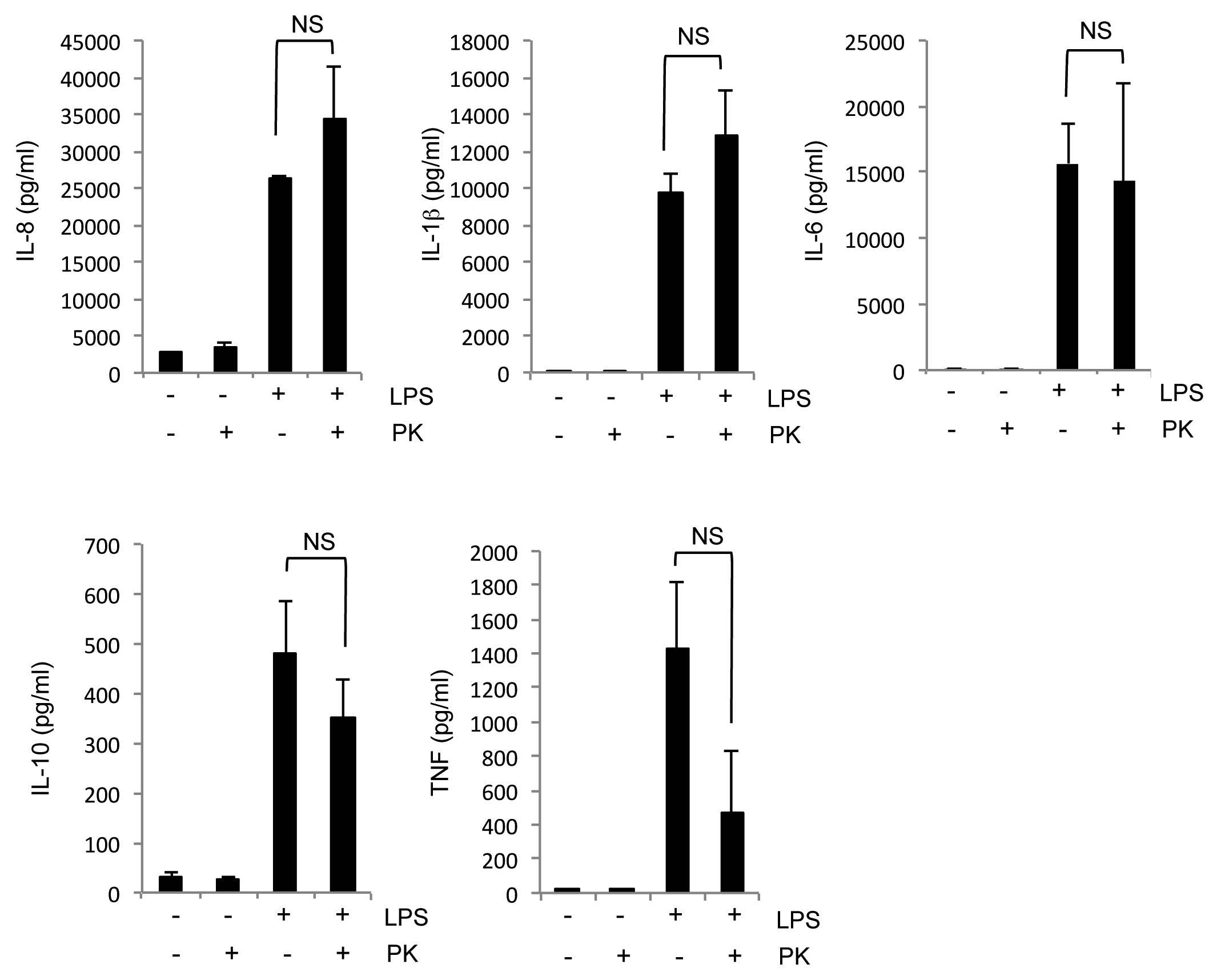

Supplement: Figure S2 — Proteinase-K does not significantly abolish LPS-induced cytokine release. LPS was treated with proteinase-K agarose beads for 30 min at 37°C before removal of proteinase-K beads. Expression of cytokines after stimulation with proteinase-K treated LPS in IL-8, IL-1β, IL-6, IL-10 and TNF in human PBMCs. Data represent the mean ±SEM of triplicate samples, results are representative of three independent experiments). * = p<0.05, ** = p<0.001; unpaired, two-tailed Student's t-test. (TIF) [file pone.0109596.s002.tif]
